# Identification of Novel Candidate Genes for Familial Thyroid Cancer by Whole Exome Sequencing

**DOI:** 10.3390/ijms24097843

**Published:** 2023-04-25

**Authors:** Cristina Tous, Carmen Muñoz-Redondo, Nereida Bravo-Gil, Angela Gavilan, Raquel María Fernández, Juan Antiñolo, Elena Navarro-González, Guillermo Antiñolo, Salud Borrego

**Affiliations:** 1 Department of Maternofetal Medicine, Genetics and Reproduction, Institute of Biomedicine of Seville, University Hospital Virgen del Rocío/CSIC/University of Seville, 41013 Seville, Spain; cristinab.tous@juntadeandalucia.es (C.T.); carmen.munoz.redondo@juntadeandalucia.es (C.M.-R.); nereida.bravo@juntadeandalucia.es (N.B.-G.); amgavilan-ibis@us.es (A.G.); raquelm.fernandez.sspa@juntadeandalucia.es (R.M.F.); jantinolob@gmail.com (J.A.); 2 Center for Biomedical Network Research on Rare Diseases (CIBERER), 41013 Seville, Spain; elena.navarro.sspa@juntadeandalucia.es; 3 Department of Endocrinology and Nutrition, University Hospital Virgen del Rocío, 41013 Seville, Spain

**Keywords:** next-generation sequencing, whole exome sequencing, thyroid cancer, hereditary cancer

## Abstract

Thyroid carcinoma (TC) can be classified as medullary (MTC) and non-medullary (NMTC). While most TCs are sporadic, familial forms of MTC and NMTC also exist (less than 1% and 3–9% of all TC cases, respectively). Germline mutations in *RET* are found in more than 95% of familial MTC, whereas familial NMTC shows a high degree of genetic heterogeneity. Herein, we aimed to identify susceptibility genes for familial NMTC and non-*RET* MTC by whole exome sequencing in 58 individuals belonging to 18 Spanish families with these carcinomas. After data analysis, 53 rare candidate segregating variants were identified in 12 of the families, 7 of them located in previously TC-associated genes. Although no common mutated genes were detected, biological processes regulating functions such as cell proliferation, differentiation, survival and adhesion were enriched. The reported functions of the identified genes together with pathogenicity and structural predictions, reinforced the candidacy of 36 of them, suggesting new loci related to TC and novel genotype–phenotype correlations. Therefore, our strategy provides clues to possible molecular mechanisms underlying familial forms of MTC and NMTC. These new molecular findings and clinical data of patients may be helpful for the early detection, development of tailored therapies and optimizing patient management.

## 1. Introduction

Thyroid cancer (TC) is the most common endocrine malignancy [1], with the global incidence rate increasing substantially over the past four decades [2]. TC arises from the accumulation of genetic mutations in parafollicular or follicular cells. The most common type of TC derives from follicular cells and is known as non-medullary thyroid cancer (NMTC) [1], although TC may also arise from the parafollicular calcitonin-producing C cells leading to medullary thyroid carcinoma (MTC), accounting for 5% of all cases [3]. Familial forms of MTC have been reported in the context of multiple endocrine neoplasia type 2 (MEN2) syndrome, representing 25% of all MTC cases. MEN2 syndrome includes the following clinical entities: MEN2A (MTC related to pheochromocytoma and/or hyperparathyroidism), MEN2B (MTC associated with marfanoid features and occasionally pheochromocytoma) and FMTC (familial medullary thyroid carcinoma only), which may be considered a variant of MEN2A [3]. In 1993, it was found that germline gain-of-function mutations in the *RET* proto-oncogene, which encodes a receptor tyrosine-kinase expressed in the derivatives and tumors of neural crest origin, are the genetic cause of 98% of MEN2A and ~95% of FMTC families [3,4], while the remaining 2–5% of clinically MEN2A and FMTC families do not carry *RET* mutations [5,6]. The genetic basis of those cases referred to as familial “non-*RET*” MTC remains to be established.

On the other hand, NMTC is histologically classified into four groups: papillary TC (PTC), follicular TC (FTC), poorly differentiated TC (PDTC) and anaplastic TC (ATC). A familial origin of NMTC (FNMTC) is suspected in 5–15% of NMTC cases by the occurrence of the disease in two or more first-degree relatives together with the absence of predisposing environmental factors [7]. Among FNMTC cases, PTC is the most common histological subtype (85–91%) reported, followed by PDTC (2–15%), FTC (6–9.7%) and ATC (1.6%) [8]. It is noteworthy that in 10-20% of sporadic cases of PTC there are RET rearrangements (RET-PTC), which seem to be related to an early event in carcinogenesis [9].

FNMTC may present both as an autosomal-dominant trait with incomplete penetrance and variable expression [10] or as a polygenic condition probably associated with low-penetrant alleles [11] and a high degree of genetic heterogeneity. To identify susceptibility genes, linkage-based strategies have been applied to large pedigrees. In this way, several genomic regions, such as FNMTC predisposing loci, have been reported [12,13,14,15,16,17,18]. So far, a few FNMTC predisposing genes have been identified, some of them located in previously susceptibility loci: *SRGAP1* [19], *DICER1* [18], miR-886-3p and miR-20a [20], *SRRM2* [21], *MYO1F* [15], *AK023948* [22], *MAP2K5* [23], *NOP53* [24], *FOXE1* [25], *PTCS2* [26], *MYH9* [27], *NKX2-1* [28], *DIRC3* [29], *CHEK2* [30], *TINF2* [31] and *POT1* [32]. These genes encode for a wide variety of proteins and cell functions, indicating that different hits may converge in this type of cancer. However, despite the solid evidence for the heritability of NMTC, only a few variants have been faithfully associated with an increased risk for NMTC development [33].

Here, we report 40 patients affected by PTC or MTC belonging to 18 families and 69 nonaffected relatives. Whole exome sequencing (WES) was applied to identify a germline variant associated with an increased PTC or MTC predisposition in those families. We believe that our findings may help in familial NMTC and MTC diagnosis in the future, improving cancer prevention and genetic counselling protocols. 

## 2. Results

### 2.1. Identification of Candidate Variants by Whole Exome Sequencing

**Figure 1:**
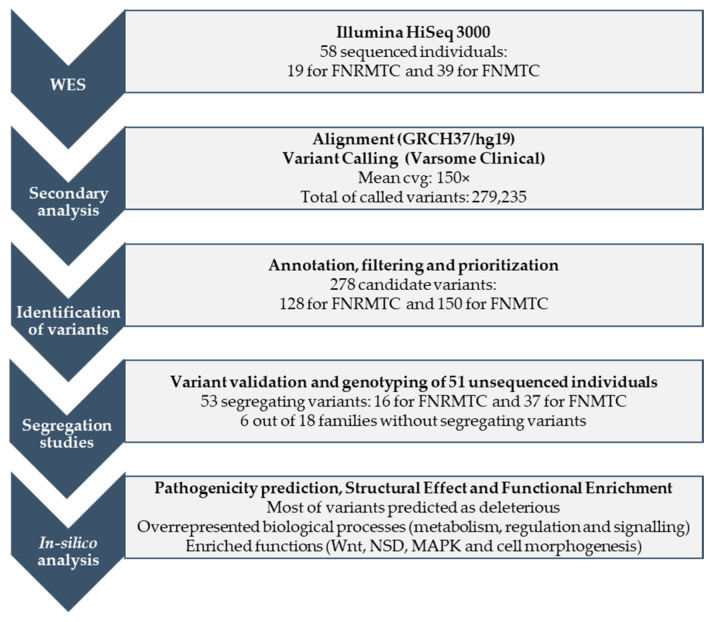
Flow chart showing the pipeline used in this study and the main outcome obtained. First, whole exome sequencing (WES) was applied to 58 individuals, 19 of them belonging to families with familial non-RET medullary thyroid carcinoma (FNRMTC) and 39 of them corresponding to familial non-medullary thyroid carcinoma (FNMTC) cases. After sequencing and primary analysis in the HiSeq 3000 platform, secondary analysis was conducted. In this step, reads were aligned to the reference human genome (GRCH37/hg19) and variants were called with the Varsome Clinical software, obtaining a mean coverage (cvg) of 150× and a total of variants of 279,235. The annotation, filtering and prioritization of these detected variants allowed for the identification of 278 candidate variants, 128 of them found in FNRMTC cases and 150 in FNMTC families. All these candidate variants were validated and segregated in all available family members, obtaining a total of 53 variants segregating with the disease in 12 out of 18 families under study. Finally, in silico analyses were performed to predict the pathogenicity and structural effect of the identified variants, as well as the functional enrichment of the mutated genes. These tools revealed that most of the segregating candidate variants were predicted as damaging and that several biological processes and functions were overrepresented and enriched, respectively. MAPK: MAPK pathway; NSD: nervous system development; Wnt: Wnt signaling pathway.

To identify genetic variants associated with familial NMTC and non-RET MTC, WES analysis was performed in 58 individuals by an in-house pipeline (Figure 1). On average, we obtained 51 million reads per sample (ranging from 40 to 62 million reads), an average read depth of 150× per base and a mean base call quality of 1379. 

After the alignment of the reads, an average of 279,235 variants were called per individual (range: 166,962–377,719). Bioinformatics filtering led to the detection of 128 candidate variants for familial non-*RET* MTC patients and 150 candidate variants for FNMTC patients, belonging to 5 and 13 families, respectively (Table 1). Segregation analysis was performed by Sanger sequencing in all family members with available DNA, which resulted in 16 variants segregating with the disease in familial non-RET MTC and 37 variants in FNMTC (Table 2). Remarkably, most segregating variants are extremely rare and were absent in the gnomAD data set (v2.1.1) [34].

### 2.2. Prioritization of Variants in Genes Previously Associated with TC

A bioinformatic filter was applied to restrict the analysis to variants within the 417 known genes previously related to familial papillary or medullary thyroid cancer and reported in the DisGeNET database (https://www.disgenet.org/, accessed on 19 February 2023) (v7.0) [35]. This analysis revealed that seven of the novel identified variants were located in genes previously associated with thyroid cancer: *MSH6*, *FOXM1*, *EPCAM*, *HOOK3*, *BMP1*, *TG* and *NTRK1*. Those variants were manually curated and classified following the American College of Medical Genetics and Genomics (ACMG) guidelines, finding four variants of uncertain clinical significance (VUS), one likely pathogenic (LP) and two pathogenic variants (Table 2).

The abovementioned genes have been described as being involved in different molecular pathways related to cell growth (*MSH6*, *FOXM1*, *EPCAM*, *HOOK3*, *BMP1*, *NTRK1* and *TG*) and cell damage (*MSH6*, *FOXM1* and *NTRK1*). The ToppFun FEA, relative to diseases, predicted a possible interaction with the proteins SMC2 (structural maintenance of chromosomes 2) and MRN complex (MRE11, RAD50 and NBN) (Table 3) [36]. 

### 2.3. Interpretation of Variants, Pathogenicity and Structural Effect Prediction 

A total of 46 segregating variants were located in genes not previously associated with TC. To assess their pathogenicity, we used 7 in silico prediction scores (CADD, PolyPhen, SIFT, LofTool, MutationAssessor, MutationTaster and FATHMM), finding 38 variants predicted as damaging in at least 60% of tools in which the prediction value was obtained (Table 4). These variants were located in 38 different genes and identified in 12 out of the 18 families analyzed in this study (Table 4). 

Metascape analysis [37], for the 45 variants predicted as deleterious (Table 4), determined that the corresponding mutated genes are implicated in vascular smooth muscle contraction (*ITPR1*, *MYH10*, *PRKG1*, *DGKQ*, *EpCAM* and *JMJD1C*), signaling by receptor tyrosine kinases (*COL4A4*, *ITPR1*, *NTRK1* and *PTPRS*), regulation of Ras protein signal transduction (*FOXM1*, *NTRK1* and *ROBO1*), regulation of canonical Wnt signaling pathway (*TNKS*, *NKD1* and *SHISA6*) and regulation of nervous system development (*NTRK1*, *PTPRS*, *ROBO1*, *TG*, *HOOK3*, *PRKG1*, *AATK* and *CCDC134*). These biological processes regulate cellular functions related to tumor initiation and progression, cell proliferation, differentiation, survival and adhesion.

Using the HOPE in silico tool (Have (y)Our Protein Explained, https://www3.cmbi.umcn.nl/hope/, accessed on 23 February 2023), it was possible to predict whether different variants affect protein stability, activity or folding. A change in size, charge and/or hydrophobicity was often observed for the introduced mutant residue (Supplementary Materials Table S1). In some cases, we observed that the mutated residue was located in a domain of the protein, which could disturb the core structure of the affected domain and alter the protein function (Table 5).

**Figure 2:**
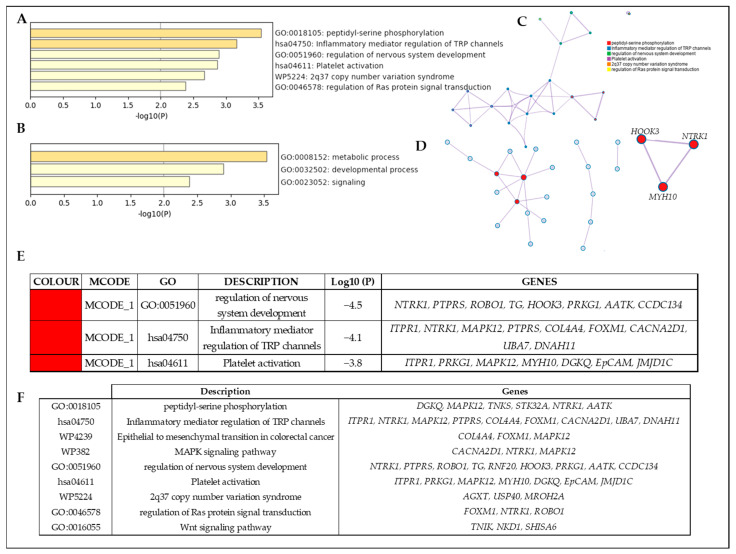
Functional enrichment analysis by Metascape. (**A**) Bar chart of clustered enrichment ontology categories (GO and KEGG terms). (**B**) The top-level Gene Ontology biological processes. (**C**) Enrichment ontology clusters including the 53 identified genes. Each term is represented by a circle node, where its size is proportional to the number of input genes falling into that term, and its color represents the cluster identity (i.e., nodes of the same color belong to the same cluster). Terms with a similarity score > 0.3 are linked by an edge (the thickness of the edge represents the similarity score). The network is visualized with Cytoscape (v3.1.2), with a “force-directed” layout and edge bundled for clarity. (**D**) Protein–protein interaction (PPI) network and the most significant MCODE components form the PPI network. (**E**) Independent functional enrichment analysis of MCODE components. (**F**) Gene enrichment in each GO cluster.

No common variants or genes were found among the 18 families analyzed by WES. However, candidate genes found in different families have been described to present similar functions. In this regard, nine of the genes with candidate variants segregating with disease in the families belong to seven different gene families whose function is related to the regulation of nervous system development (GO:0051960) (*NTRK1*, *PTPRS*, *ROBO1*, *TG*, *RNF20*, *HOOK3*, *PRKG1*, *AATK* and *CCDC134*) or inflammatory mediator regulation of TRP channels (*ITPR1*, *NTRK1*, *MAPK12*, *PTPRS*, *COL4A4*, *FOXM1*, *CACNA2D1*, *UBA7* and *DNAH11*) (Figure 2F).

### 2.4. Functional Enrichment Analysis Identified the Most Relevant Genes and Gene–Gene Modules

To better understand the biological context of the selected candidates, we performed a gene ontology (GO) term enrichment analysis for the selected genes using the Metascape software tool [37] (Figure 2). We found overrepresented GO-Biological Process (GO-BP) terms (Figure 2A), and 25 out of 53 genes were included in a biological network (Figure 2C). The biological process analysis showed that most of the genes were engaged in “metabolic process”, “biological regulation” and “signaling” (Figure 2B). Regarding the molecular function categories, “peptidyl-serine phosphorylation”, “regulation of nervous system development” and “inflammatory mediator regulation of TRP channels” captured the principal functions. The GO functional enrichment analysis showed that the candidate variants were mainly enriched in the Wnt signaling pathway (*TNKS*, *NKD1* and *SHISA6*), regulation of nervous system development (*NTRK1*, *PTPRS*, *ROBO1*, *TG*, *HOOK3*, *PRKG1*, *AATK* and *CCDC134*) or regulation of Ras protein signal transduction, including the MAPK pathway (*CACNA2D1*, *NTRK1* and *MAPK12*), or cell morphogenesis (*NTRK1*, *PRKG1* and *ROBO*). The molecular complex detection (MCODE) enrichment analysis based on protein–protein interactions (PPIs) enrichment analysis resulted in a network characterized by the presence of one PPI module, including the genes *HOOK3*, *MYH10* and *NTRK1* (Figure 2D). Among the top list of enriched terms of Module 1, we found three major GO-BP clusters: regulation of nervous system development, inflammatory mediator regulation of TRP channels and platelet activation (Figure 2E). 

### 2.5. Immunohistochemistry Analysis of Available PTC Samples

**Figure 3:**
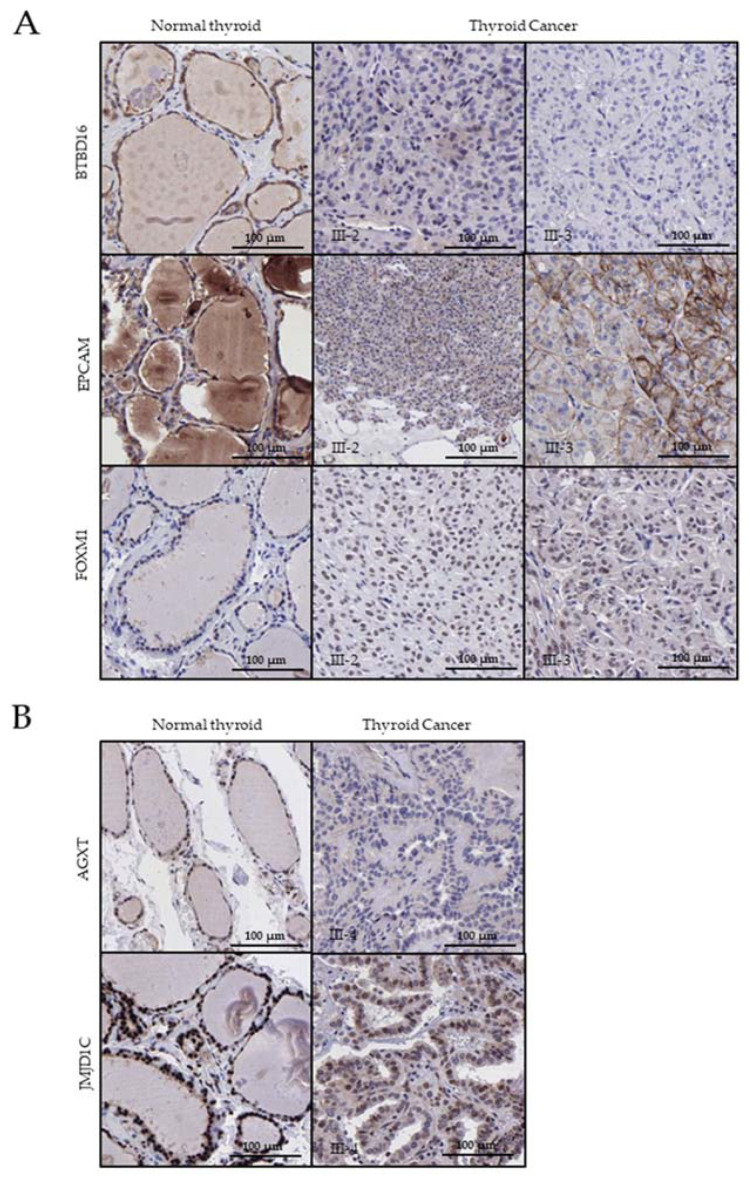
Immunohistochemical detection of BTBD16, EpCAM, FOXM1, AGXT and JMJD1C expression in normal and tumor thyroid tissue samples of three patients from two NMTC families: (**A**) NMTC_1 and (**B**) NMTC_4. Original magnification = ×20. Scale bar = 100 µm.

Immunohistochemistry (IHC) staining for BTBD16, EpCAM, FOXM1, AGXT and JMJD1C was carried out in normal and tumor thyroid tissue samples (three patient biopsies available), belonging to two NMTC families: NMTC_1 (Figure 3A) and NMTC_4 (Figure 3B). We considered performing immunochemistry assays for the six mutated genes segregating in these families, although the KRT39 experiment could not be conducted.

Specific immunolabeling with the BTBD16 and AGXT antibodies was detected in control samples, while a lack of expression of these genes was found in tumoral tissues. On the other hand, the expression of EpCAM and FOXM1 was increased in tumoral tissue in comparison with the controls. It is worth noting that EpCAM expression can be appreciated in the membrane in normal conditions, while, in tumoral thyroid tissue, it is located at the cytoplasm and membrane (Figure 3A), the cytoplasmatic expression being more intense in patient III-2 than patient III-3. Concerning JMJD1C, we observed a decreased expression pattern with respect to normal thyroid tissue.

## 3. Discussion

Familial thyroid carcinomas can be either MTC or NMTC. Five percent of familial forms of MTC are not explained by an autosomal-dominant gain-of-function mutation in the *RET* proto-oncogene (familial non-*RET* MTC). Additionally, the susceptibility genetic background for familial NMTC is still unknown, although currently emerging. In this study, we aimed to identify genetic variants involved in familial non-*RET* MTC and NMTC using WES in 58 individuals belonging to 18 Spanish families. This strategy resulted in the identification of a total of 53 variants segregating with the disease, 36 of which were considered candidates according to in silico predictions and the reported gene function.

First, focusing exclusively on MTC, we detected 16 rare variants segregating in four out of five families (Table 1). No candidate variants were found in family MTC_2, and none of the variants or mutated genes identified in the remaining MTC families were shared. Therefore, we could not identify a common molecular cause in the studied MTC cases. However, in three out of five MTC families (MTC_1, MTC_4 and MTC_5), we found pathogenic or likely pathogenic variants located in genes involved in biological functions crucial for tumor development, such as cell proliferation.

Cell proliferation is a critical issue for either normal development or tumor growth. More than one signaling pathway has been involved in the regulation of cell proliferation. For example, the Wnt/β-catenin signaling, PI3K/mTOR pathway, NFκB signal, MAPK pathway and other signaling pathways all regulate cell proliferation and cross with each other to form a signal network [38,39]. In family MTC_1, we identified the variant c.3548A>G in the *PTPRS* gene. This variant was predicted as deleterious for most of the used in silico tools (six out of seven, including CADD) and, according to HOPE results, the amino acid change could cause a defective protein as the hydrophobicity and size would be increased and interactions would be affected (Supplementary Materials Table S1). The *PTPRS* gene codifies to a tyrosine-protein phosphatase, whose function is to modulate ERK phosphorylation and prevent translocation to the nucleus [40]. The physiological role of this gene is well established in the nervous system and pituitary development [40]. Mutation or deletion of *PTPRS* has been reported in several human cancers, including head and neck squamous cell carcinoma [41], colorectal cancer [42], malignant melanoma [43] and cholangiocarcinoma [44]. *PTPRS* silencing promoted cell proliferation, migration and invasion [45]. PTPRS is an important gene in regulating the RAS/ERK pathway, although their functional role in cancer is much less understood than their counterpart protein tyrosine kinases. The cumulative results of predictors and structural protein indicate that selected tumor-associated *PTPRS* mutation is a strong candidate to be associated with the development of non-*RET* MTC, but mutational screening of this gene in these familial cases should be clarified.

In family MTC_4, we detected nine segregating variants in the genes *UBA7*, *NICN1*, *MROH2A*, *IL16*, *DDX51*, *CCDC134*, *ANKRD24*, *DNAH11* and *MAPK12*. The variant of the NICN1 gene was the only one found in this family for which both of the most pathogenicity predictors, the HOPE resource and the known gene function, were consistent and supported a likely deleterious effect on the mutated protein and its involvement in the development of TC. This gene is essential for controlling microtubule functions in different cell types and organelles, which has been linked to ciliopathy [46], cancer [47,48] and neurodegeneration [49]. Although the variants of *IL16*, *CCDC134* and *DNAH11* were also predicted as likely pathogenic for most of the prediction tools, no alterations were detected by HOPE analysis. This makes sense considering that these three variants are truncated, since these types of variants tend to be predicted as pathogenic by default, even though it has not necessarily been so. The literature and database review showed that these three genes could also be related to the development of cancer, either by playing an indispensable role in the activation of the MAPK pathway (in the case of *DNAH11*) [50] or, as immune cytokines, in the case of *CCDC134* and *IL16*, affecting the signaling activity that facilitates tumor progression [51,52]. Otherwise, the pathogenicity of variants found in *UBA7*, *MROH2A*, *DDX51* and *MAPK12* were not seconded by in silico predictors, but the HOPE results indicate a possible effect on the protein features, since the mutated residues were located in protein domains relevant for their activities and interactions. The exhaustive bibliographic search highlighted that three of these genes presented functions associated with tumor development, including relationships to the canonical Wnt signaling for *DDX51* [53], the MAPK pathway for *MAPK12* [54] and the post-translational modifications activating an antitumor gene expression program for *UBA7* [55]. In contrast, we have not found sufficient bibliographic evidence to support the role of *ANKRD24* and *MROH2A* in the tumor process. Therefore, the results obtained for this family have not allowed us to conclude which variant or set of variants may be responsible for the disease, and additional studies to continue studying this case would be needed, prioritizing the *NICN1* variant together with the rest of the novel variants located in genes functionally related to cancer (*UBA7*, *IL16*, *DDX51* and *CCDC134*).

In the analysis of the MTC_5 family, we found that five variants in the genes *ZNF19*, *USP40*, *DGKQ*, *COL4A4* and *MSH6* segregated with the disease (Table 1). Whereas the *MSH6*, *DGKQ* and *COL4A4* variants were predicted as damaging for most of the in silico prediction tools (Table 4), the structural analysis highlighted the variants of the ZNF19 and COL4A4 proteins due to the fact of both producing a change in the charge of the protein affecting to a structurally important protein region (Supplementary Materials Table S1). Remarkably, *MSH6* is a tumor suppressor gene previously known to be implicated in the development of TC [56]. Specifically, the p.Arg1076Cys MSH6 mutation has been previously reported as pathogenic for Lynch syndrome [57] and various types of cancer, such as lung [58], pancreatic [59] and breast cancer [60]. The remaining detected variants were also located in genes with functions related to tumor processes, including transcriptional regulation and interaction with the Trim28 protein (*ZNF19*) [61], regulation of p53 and apoptosis (*USP40*) [62], induction of the Ras-MAPkinase/ERK and Akt/PKB pathways (*DGKQ*) [63] and cell proliferation (*COL4A4*) [64]. However, the USP40 variant did not fit the established criteria for in silico tools to be considered as a final candidate. Thus, the occurrence of TC in this family is likely to be caused, at least in part, by the *MSH6* mutation, while the involvement of the rest of the detected variants should be further studied.

In the last MTC family with detected variants (MTC_3), the identified change was predicted as damaging for four out of seven prediction tools. This variant led to a mutation in serine 621, which is located very close to S588 and T642, sites of phosphorylation by AKT kinase and necessary for the functionality of the protein. In fact, the structural analysis showed that the protein could be affected by this variant in size and charge, as well as in hydrophobicity, interactions and folding. The *TBC1D4* gene is a Rab-GTPase activator protein involved in glucose transporter 4 (GLUT4) [65]. The expression of GLUTs has been found in different cancers to modulate glucose metabolism and correlate with epithelial–mesenchymal transition (EMT) [66], chemotherapy resistance [67] and cell proliferation [68]. Therefore, at present, we do not have sufficient evidence to propose this variant as a candidate for the development of TC in this family and additional studies would be needed.

On the other hand and regarding familial forms of NMTC, we identified 37 rare variants segregating with the disease in 8 out of 13 FNMTC families included in this study. In silico tools showed that 25 of the identified variants were predicted as deleterious for most of the predictors used and, therefore, could be considered candidate susceptibility variants for TC (Table 4).

In 62.5% (five out of eight) of the NMTC families with segregating variants, we found a total of 6 variants located in genes previously associated with thyroid cancer (Table 2). All of these variants were novel and predicted as deleterious for most of the in silico prediction tools. Moreover, according to the HOPE project, variants in *TG* (NMTC_7), *NTRK1* (NMTC_11) and *BMP1* (NMTC_12) could be affecting a specific domain of the protein (Table 5), while those in *FOXM1* (NMTC_1), *EpCAM* (NMTC_1) and *HOOK3* (NMTC_6) could be altering the charge and size of the mutated amino acid (Supplementary Materials Table S1). In addition, *EpCAM* and *TG* variants were classified as likely pathogenic and pathogenic, respectively, by ACMG criteria, while the remaining variants were considered uncertain. Nevertheless, their null population frequency, family segregation and the in silico results suggest that these variants are likely to be causative for the TC of the corresponding families, although the degree of involvement of each of them would still be determined and the role of the additional variants found in these families should not be overlooked.

In this light, in family NMTC_1, we found two other variants in genes not previously associated with TC (*KRT39* and *BTBD16*). Both variants led to a truncated protein for which the obtained outcome of in silico tools was very limited. The IHC study (Figure 3) showed an altered expression for the three genes in the patient samples compared to control samples, reinforcing the involvement of *EpCAM* and *FOXM1* in the TC of this family and proposing *BTBD16* as a strong candidate TC-gene. Whereas overexpression of *FOXM1* caused by the identified mutation could induce cell proliferation and, consequently, TC since its silencing blocks growth, migration and invasion of PTC cells [69], the high expression of *EpCAM* could indicate a poor prognostic because it involves modulating biological processes such as cell proliferation, differentiation, migration and invasion [70]. On the other hand, the lack of expression of BTBD16 in thyroid tumor tissue would mean certain implications, but we have not found sufficient bibliographic evidence to conclude its role in TC.

It is important to note that NTRK1, the previously TC-associated gene identified in family *NMTC_11*, is an oncogene expressed in neural and nonneuronal tissues and like *RET*, is often activated by rearrangements [71]. However, to date, no germline point mutations have been described in PTC hence these results could be indicating a new genotype-phenotype correlation. Furthermore, in this family, the *TNKS* and *PPP6R2* variants could be candidates to accompany the *NTRK1* mutation at the onset of the disease. While PPP6R2 is a regulatory protein implicated in cell cycle entry and progression whose loss of function causes genomic instability [72], TNKS is involved in the Wnt pathway whose destabilization and deregulation led to uncontrolled proliferation and, therefore, progression of cancers [73]. There is evidence that thyroid carcinomas are dependent on Wnt signaling for growth and proliferation [74]. In fact, we found other variants (*SHISA6*, *GGNBP2* and *NKD1*) affecting different levels of the Wnt pathway, according to GO functional (Figure 2F), in additional studied NMTC families (NMTC_2, NMTC_6 and NMTC_7, respectively), which could also be an explanation for the development of TC in their corresponding families, either alone or together with other susceptibility variants detected in this study or even pending identification.

As previously mentioned, among these families with mutated genes related to the Wnt pathway, we find the NMTC_7 family. In this family, in addition to the strong candidate variant in *NKD1* and the pathogenic mutation in a functional domain of the TC-gene *TG* [75], we also found four variants in the genes *ROBO1*, *MYH10*, *CSMD2* and *STK32A*, all of them with a positive expression in thyroid cancer according to public databases, suggesting that they could play a role in familial NMTC development. Whereas changes in the expression of the *MYH10*, *CSMD2* and *STK32A* have been reported in other tumors causing alterations in cell proliferation [76,77,78], the gene *ROBO1* has a significant association with the regulation of nervous system development [79]. It is noteworthy that, beyond the variant in the Wnt-related gene *GGNBP2*, two other variants were found in the NMTC_6 family, which were also implicated in this biological function (Figure 2F): *RNF20*, an E3 ligase of H2BK120 regulating astrocyte production from neural precursor cells [80] and *HOOK3*, which mediates binding between microtubules and organelles, important for maintaining genomic integrity and for the maintenance of proper centrosome function in neural cells [81,82].

The last family in which variants were identified was the NMTC_12 family. In addition to the highly thyroid-expressed *BMP1* gene, candidate variants were also detected in *ITPR1*, *PRKG1*, *DENND2B* and *THSD7A*. Whereas variants of *DENND2B* and *THSD7A* involve changes in their structure that lead to a decrease or increase in their expression to promote uncontrolled cell growth [83,84], the variant in *PRKG1* could be affecting the catalytic domain of this protein kinase. A review of the literature revealed previous reports of the involvement of PRKG1 in the apoptosis of cancer cells via hyperactivation of death-associated protein kinase 2 [85]. The *ITPR1* variant could be altering the inflammatory and immune response involved in the pathogenesis of conditions such as cancer since this function is enriched in the MetaScape analysis (Figure 2F) by this and other candidate genes (*FOXM1*, *CACNA2D1* and *NTRK1*). Interestingly, in the GO functional enrichment analysis, we also found *CACNA2D1*, together with *NTRK1*, implicated in the MAPK pathway (Figure 2F) [71]. Elevated *CACNA2D1* expression has been reported to be associated with poor prognosis in several types of cancer [86,87,88,89]. This gene, as well as the Wnt-related gene *SHISA6*, was mutated in family NMTC_2, in which a third candidate variant was also detected in the *AATK* gene, associated with apoptosis [90]. Loss of AATK expression is observed in multiple tumor entities which drive to uncontrolled cell growth by interfering with a signaling cascade including TP53 phosphorylation and reduction in expression of the key cell cycle regulators CCND1 and WEE1 [91].

In the NMTC_4 family, mutations in two different genes (*JMJD1C* and *AGXT*) were identified and, in both cases, the variant led to a change in the wildtype (wt) amino acid for one larger and introduce a positive charge (Supplementary Table S1). In the IHC analysis of tumor tissue from the affected member of this family, we observed a lack of expression of *AGXT* in tumor tissue, which could be indicating a loss-of-function (LOF) of the protein. However, the IHC results for the *JMJD1C* gene showed a lower protein expression than normal thyroid tissue (Figure 3B). *JMJD1C* functions as histone demethylases but may possess other roles that act independently of the JmjC domain, such as regulating epigenetic patterns and interactions [92] with the thyroid hormone receptor (TR) [93]. These functional results further support the accurate filtering here performed and the candidacy of the candidate genes and variants identified in this study, although additional studies are still needed to conclude the involvement of each of them in the development of TC. Finally, larger-scale studies should be carried out in families where no candidate variants were detected, always keeping in mind the possibility that the underlying genetic defect is not identifiable by the current technology.

## 4. Materials and Methods

### 4.1. Study Participants

**Figure 4:**
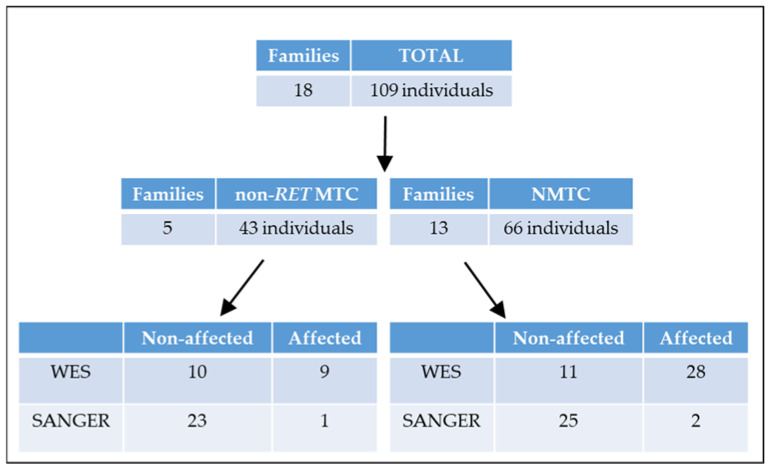
Diagram showing the cohort of patients used in this study. non-*RET* MTC: non-*RET* medullary thyroid carcinoma; NMTC: non-medullary thyroid cancer; SANGER: Sanger sequencing; WES: whole exome sequencing.

All patients included in this study were referred to the Department of Maternofetal Medicine, Genetics and Reproduction from the University Hospital Virgen del Rocío (Seville, Spain). In total, 18 families were studied in this survey (Supplementary Materials Figures S1 and S2), including 40 patients and 69 unaffected family members, making a total of 109 individuals. Five of the studied families have a non-*RET* medullary thyroid cancer phenotype, whereas thirteen families have papillary thyroid cancer (Figure 4). All affected members belonging to each family were screened and discarded for mutations in the exons of the *RET* proto-oncogene.

Genomic DNA was isolated after peripheral blood extraction using standard procedures. DNA integrity was assessed before performing each of the NGS methods using spectrophotometric and fluorometric dsDNA quantification and 1% agarose gel electrophoresis. 

### 4.2. Whole Exome Sequencing (WES)

To identify genetic variants associated with familial forms of non-*RET* MTC and NMTC, WES analysis was performed on at least two TC cases and one unaffected relative per family when available, resulting in 58 sequenced individuals (37 affected and 21 unaffected) (Figure 4 and Supplementary Materials Figures S1 and S2). Thirty-nine of these sequenced individuals (28 diagnosed with FNMTC and 11 healthy relatives) belonged to the 13 NMTC families, while nineteen of them (9 patients and 10 unaffected subjects) belonged to the 5 MTC families (Figure 3).

The DNA samples were sheared by sonication (Covaris M220, Covaris Inc., Woburn, MA, USA), repaired and ligated following the Accel-NGS 2S DNA library kit, DL-IL2SH-48 (Swift Bioscience, Ann Arbor, MI, USA). Pre-amplification and addition of dual-index steps were conducted by using the KAPA HiFi hot-start ready mix, KK2602 (Kapa Biosystems, Wilmington, MA, USA). Fragments ranging in size from 200 to 400 bp were selected by beads (Agencourt AMPure XP Kit, A63880, Beckman Coulter, Pasadena, CA, USA), followed by hybridization with the capture probes (Integrated DNA Technologies, Coralville, IA, USA). Probe–DNA hybrids were recovered and purified using magnetic beads. Quality control of the library was performed on Agilent 2100 bioanalyzer (Agilent Technologies, Inc., Waldbronn, Germany). The indexed library was pooled for loading onto flow cells for sequencing on the HiSeq3000 platform (Illumina, San Diego, CA, USA) using a HiSeq 3000/4000 SBS Kit (300 cycles) and a HiSeq 3000/4000 PE Cluster Kit. Raw image files were produced by calling with default parameters, and the sequences generated by paired-end reads.

### 4.3. Bioinformatics Analysis

The data analysis was performed using the VarSome Clinical platform (version 11.6) [94]. The reads, in FASTQ format, were loaded into the platform and aligned to the reference human genome (hg19) using the Sentieon aligner (bwa-mem), while variant calling for SNVs and small indels was performed using the Sentieon GATK caller [95]. Variants were classified as pathogenic, likely pathogenic, variants of uncertain significance (VUS), likely benign and benign, according to the American College of Medical Genetics and Genomics (ACMG) guidelines [96] and the Association for Clinical Genomic Science (ACGS) recommendations [97], using the help of the ACMG automated classifier of Varsome (version 11.6). 

The variants were filtered according to the next steps: (a) common variants with a MAF > 0.001 were excluded; (b) synonymous and noncoding variants were removed, except variants located in splicing regions spanning up to 20–30 bases of each exon; (c) variants with an allelic fraction lower than 30% were discarded; (d) variants with a CADD phred score ≤ 20 and/or with an ACMG classification ≤ 3 were filtered out; and (e) heterozygous or homozygous variants shared by all affected patients were prioritized, while variants present in more than one unaffected individual with a genotype compatible with the inheritance pattern of the disease were eliminated (pedigree filtering). It is worth mentioning that for those variants that Varsome does not provide a value for CADD, the Variant Effect Predictor (www.https://www.ensembl.org/Tools/VEP) [98], public Galaxy platform (www.usegalaxy.org) [99] and CADD (https://cadd.gs.washington.edu/) [100] tools were used to obtain this value.

According to the MAF filtering, it is noteworthy that the initial filter consisted of prioritizing only variants with not frequency data (MAF = 0). However, as no candidate variants were detected in 6 families, a more relaxed frequency filter (≤0.001) was finally applied.

The next additional information was also checked for the remaining SNVs and small indels, and used for their final interpretation: (a) in silico prediction scores to evaluate the potential impact and pathogenicity of the candidate variants on the function or structure of the protein and conservation by SIFT [101], PolyPhen2 [102], Mutation Taster [103], LofTool [104], MutationAssessor [105] and FATHMM [106]; (b) analysis of the physiochemical changes induced by the variants using the server HOPE (http://www.cmbi.ru.nl/hope) [107]; (c) the clinical significance of variants according to public and private databases, such as ClinVar (https://www.ncbi.nlm.nih.gov/clinvar/) and HGMD Professional version 2022.4 (http://www.hgmd.org; and (d) the reported gene function, location and expression, as well as its relationship to known pathological mechanisms of TC, according to The Human Protein Atlas (https://www.proteinatlas.org/), OncoMX (https://www.oncomx.org/), KEGG Pathway Database (https://www.genome.jp/kegg/pathway.html) and the literature.

### 4.4. Validation of Genetic Variants

Following data filtering, the identified causative variants were confirmed by conventional Sanger sequencing in DNA samples of the patients and their parents. For this purpose, specific primers were according to in-house pipelines, making use of the IDT online software (PrimerQuest™ program, IDT, Coralville, IA, USA. accessed 12 December 2018. https://www.idtdna.com/SciTools). Primer sequences and annealing temperatures can be found in Supplementary Materials Table S2. The purified PCR products were sequenced on an ABI 3730xL instrument (Applied Biosystems, Waltham, MA, USA) using the BigDye 3.1 Terminator sequencing kit (Applied Biosystems) according to the manufacturer’s protocol. Sequences were analyzed using SeqMan NGen^®^ (Version 17.2. DNASTAR. Madison, WI, USA). 

### 4.5. Functional Enrichment Analysis, Network, and PPI Module Reconstruction

Those genes that harbored validated variants were further studied through pathway enrichment analysis and gene network reconstruction by the Metascape tool (https://metascape.org/) [108] with the default parameters set. Imputing the gene set obtained, pathway and enrichment analyses were carried out by selecting the genomics sources: KEGG Pathway, GO Biological Processes, Reactome Gene Sets, Canonical Pathways, and CORUM [109]. All genes in the genome were used as the enrichment background. Terms with *p* < 0.01, minimum count 3, and enrichment factor > 1.5 (the enrichment factor being the ratio between observed count and the count expected by chance) were collected and grouped into clusters based on their membership similarities. Subsequently, using Metascape default parameters, based on PPI enrichment analysis, we run a module network reconstruction based on the selected genomics databases. Through the MCODE algorithm, we first identified connected network components. A pathway and process enrichment analysis was applied to each MCODE component independently and the three best-scoring (by *p*-value) terms were retained as the functional description of the resulting modules.

The genes candidate related to TC were inserted in the ToppFun tool of the ToppGene database (http://toppgene.cchmc.org) [110] so as to perform Functional Enrichment Analysis (FEA) relative to Biological Process (BP) and predict possible Interactions. ToppFun performs FEA of input gene list based on the transcriptome, proteome, regulome, ontologies, phenotype, pharmacome and bibliome assuming the whole genome as background. The *p*-values were corrected for multiple testing with the Bonferroni and false discovery rate (FDR) methods of Benjamini–Hochberg and Benjamini–Yekutieli to determine statistical significance.

### 4.6. Immunohistochemistry

Formalin-fixed, paraffin-embedded (FFPE) thyroid samples from three family members affected by PTC (NMTC_1 III-2; NMTC_1 III-3 and NMTC_4 III-4) were available for immunohistochemistry (IHC) analysis.

Four-micrometer-thick tissue sections from paraffin blocks were dewaxed in xylene and rehydrated in a series of graded alcohols. Antigen retrieval was performed with a PT Link instrument (Agilent), using EDTA buffer (97 °C, 20 min). Later, sections were immersed in 3% H_2_O_2_ aqueous solution for 10 min to exhaust endogenous peroxidase activity and then covered with 1% blocking reagent (Roche) in PBS, to block nonspecific binding sites, for 20 min. Sections were incubated with FOXM1(sc-376471, 1:1000), EpCAM (MA5-12436, 1:1000), BTBD16 (PA5-59208, 1:500), JMJD1C (sc-101073, 1:1000) and AGXT (PA5-83270, 1:500) dilution overnight, except for FOXM1 (only five minutes), at 4 °C in a humid chamber. Peroxidase-labeled secondary antibodies and 3,3-diaminobenzidine were applied to develop immunoreactivity, according to the manufacturer’s protocol (Bond Polymer Refine Detection system, Leica Biosystems, Wetzlar, Germany). Slides were then counterstained with hematoxylin and mounted in DPX (BDH Laboratories, Dubai, United Arab Emirates).

## 5. Conclusions

Altogether, the obtained results reveal several common key functions among the identified mutated genes and involved in the formation of carcinomas, such as loss of cell–cell contact, dynamic changes in cell morphology, increased proliferation, and enhanced cell migration and invasion. Moreover, this study has allowed proposing 36 candidate susceptibility cancer variants in a total of 10 families (seven NMTC families and three non-*RET* MTC families). Extended family segregation for these variants could not only confirm or discard their involvement in the etiopathogenesis of TC but may assign a risk status for each family member and an appropriate level of surveillance. Determining familial risk would also allow for a prompt diagnosis in currently presymptomatic individuals, as well as offer genetic and reproductive counselling. However, it is important to note that the absence of these candidate variants is not enough to exclude individuals from a family with a positive history of TC from surveillance.

In summary, this NGS approach led us to expand the mutational spectrum of familial forms of TC, suggesting new candidate variants, genes and genotype-phenotype correlations. Collectively, these results raise new lines for the research of TC by investigating the involvement of the novel-identified loci and reinforcing the known importance of concurrent variants for the development of cancer. Furthermore, this study has also acted as a first screening step to select those negative families that may be good candidates to be analyzed by other technologies, as well as to highlight those cases in which their hereditary character should be re-evaluated.

## Figures and Tables

**Table 1:** An overview of the main outcomes of the prioritization and validation steps obtained for each of the studied families. The number of total variants resulting for each filtering step refers to an average value when several affected individuals were sequenced for the same family. The filter ACMG Class ≥ 3 refers to the classification of variants based on the ACMG criteria. All variants were nonsynonymous substitutions and small indels, resulting in missense, nonsense, frameshift and in-frame variants. All segregating variants were novel (absence of population frequency), except variants indicated with * (MAF = 0.02%) and ** (MAF = 0.1%).

	Family ID	Total Variants	Total Nonsynonymous Exonic and Splicing Variants	Total of Rare Variants MAF ≤ 0.001	Total of Rare Variants MAF = 0	Pedigree Filtering	Total Variants with CADD ≥ 20 and/or ACMG Class ≥ 3	Segregating Variants	
								Total	Genes
Familial non-RET MTC	MTC_1	287,437	26,590	1710	589	150	32	1	*PTPRS*
	MTC_2	278,892	26,647	1798	561	141	47	0	
	MTC_3	377,719	31,384	2118	635	62	10	1	*TBC1D4*
	MTC_4	331,244	27,660	1815	639	223	25	9	*UBA7* *NICN1* *MROH2A* *Il16* *DDX51* *CCDC134* *ANKRD24* DNAH11 * MAPK12 **
	MTC_5	327,240	27,595	1822	609	36	14	5	*ZNF19* *USP40* *MSH6* *DGKQ* *COL4A4*
Familial NMTC	NMTC _1	283,011	26,096	1649	539	36	17	4	*FOXM1* *EpCAM* *KTR39* *BTBD16*
	NMTC _2	295,103	26,564	1692	543	32	12	3	*CACNA2D1* *SHISA6* *AATK*
	NMTC _3	293,795	25,850	1662	549	22	11	0	
	NMTC _4	350,840	27,682	1850	602	20	8	2	*JMJD1C* *AGXT*
	NMTC_5	293,604	26,576	1726	591	45	28	0	
	NMTC_6	308,316	26,747	1643	535	34	7	3	*HOOK3* *RNF20* *GGNBP2*
	NMTC_7	166,962	23,462	1303	409	245	18	9	*NKD1* *ROBO1* *MYH10* *TTC28* *ZZEF1* *CLIC6* *CSMD2* *STK32A* *TG*
	NMTC_8	362,285	29,160	1892	633	12	4	0	
	NMTC_9	307,691	26,615	1817	529	16	6	0	
	NMTC_10	343,679	28,984	1850	571	21	4	0	
	NMTC_11	306,989	26,246	1536	499	34	12	5	*NTRK1* *TNKS* *PPP6R2* *OR51M1* *ANKRD35*
	NMTC_12	166,132	23,605	1370	461	256	13	8	*FNTB* *ITPR1* *PRKG1* *DENND2B* *BMP1* *USH2* *INC* *THSD7A*
	NMTC_13	281,709	26,292	1795	604	27	10	3	*MPPE1* *BEAN1* *KTI12*

**Table 2:** Potential susceptibility variants identified in this study. The dbSNP column corresponds to the reference SNP identifiers (rs ID) according to NCBI’s dbSNP database (v150). ACMG class refers to the variant classification following the American College of Medical Genetics and Genomics (ACMG) criteria: pathogenic (P), likely pathogenic (LP), uncertain clinical significance (VUS), likely benign (LB) and benign (B). The Combined Annotation Dependent Depletion score on the Phred scale (CADD phred) is shown for each segregating variant, for which a score of 20 means that a variant is among the top 5% of deleterious variants in the human genome. The reported clinical significance (Clin Sig.) were checked in both of the ClinVar (a) and HGMD professional (b) databases. The candidate genes considered based on their reported functions and the in silico outcomes are highlighted in grey. NA: not available.

	Family ID	Gene	Position	cDNA	Protein	dbSNP	ACMG Class	Clin Sig.	Previous TC-assoc.
Familial non - *RET* MTC	MTC_1	*PTPRS*	chr19:5220272	NM_002850.4:c.3548A>G	p.Glu1183Gly	-	LP	NA^a,b^	No
	MTC_3	*TBC1D4*	chr13:75900504	NM_014832.5:c.1862C>T	p.Ser621Leu	rs888445750	VUS	NA^a,b^	No
	MTC_4	*UBA7*	chr3:49848264	NM_003335.3:c.1232G>A	p.Arg411Lys	-	VUS	NA^a,b^	No
		*NICN1*	chr3:49466617	NM_032316.3:c.56G>A	p.Gly19Asp	-	VUS	NA^a,b^	No
		*MROH2A*	chr2:234712121	NM_001367507.1:c.1736T>C	p.Leu579Pro	-	VUS	NA^a,b^	No
		*IL16*	chr15:81585187	NM_172217.5:c.1712_1718del	p.Glu571Glyfs*7	-	LP	NA^a,b^	No
		*DDX51*	chr12:132625554	NM_175066.4:c.1262C>T	p.Pro421Leu	-	VUS	NA^a,b^	No
		*CCDC134*	chr22:42205996	NM_024821.5:c.217del	p.Leu73Serfs*29	-	VUS	NA^a,b^	No
		*ANKRD24*	chr19:4217972	NM_133475.1:c.2815G>C	p.Glu939Gln	-	LB	NA^a,b^	No
		*DNAH11*	chr7:21628196	NM_001277115.2:c.1915C>T	p.Gln639*	rs200073714	LP	NA^a^/P^b^	No
		*MAPK12*	chr22:50699625	NM_002969.6:c.226C>T	p.Arg76Cys	rs138533200	VUS	NA^a,b^	No
	MTC_5	*ZNF19*	chr16:71509902	NM_006961.4:c.548A>G	p.His183Arg	-	VUS	NA^a,b^	No
		*USP40*	chr2:234394522	NM_001365479.2:c.3299C>A	p.Ala1111Asp	-	VUS	NA^a,b^	No
		*MSH6*	chr2:48030612	NM_000179.3:c.3226C>T	p.Arg1076Cys	rs63750617	P	LP^a,b^	Yes
		*DGKQ*	chr4:955789	NM_001347.4:c.2296C>T	p.Pro766Ser	-	VUS	NA^a,b^	No
		*COL4A4*	chr2:227985771	NM_000092.5:c.286G>A	p.Asp96Asn	rs772710366	VUS	NA^a,b^	No
Familial NMTC	NMTC_1	*FOXM1*	chr12:2983469	NM_202002.2:c.176C>A	p.Pro59Gln	-	VUS	NA^a,b^	Yes
		*EpCAM*	chr2:47613735	NM_002354.3:c.928A>T	p.Arg310Trp	-	LP	NA^a,b^	Yes
		*KRT39*	chr17:39118666	NM_213656.4:c.858del	p.Trp286Cysfs*6	-	LP	NA^a,b^	No
		*BTBD16*	chr10:124045631	NM_144587.5:c.253G>T	p.Glu85*	-	VUS	NA^a,b^	No
	NMTC_2	*CACNA2D1*	chr7:81579757	NM_000722.4:c.3227A>C	p.Gln1076Pro	-	VUS	NA^a,b^	No
		*SHISA6*	chr17:11461541	NM_207386.4:c.1576C>T	p.His526Tyr	-	VUS	NA^a,b^	No
		*AATK*	chr17:79096394	NM_001080395.3:c.1342C>T	p.Pro448Ser	rs1256230088	LP	NA^a,b^	No
	NMTC_4	*JMJD1C*	chr10:64967686	NM_032776.3:c.3743A>G	p.Gln1248Arg	-	LP	VUS^a^/LP^b^	No
		*AGXT*	chr2:241815402	NM_000030.3:c.827T>G	p.Leu276Arg	-	LP	NA^a,b^	No
	NMTC_6	*HOOK3*	chr8:42841854	NM_032410.4:c.1448G>T	p.Gly483Val	-	VUS	NA^a,b^	Yes
		*RNF20*	chr9:104313988	NM_019592.7:c.1295A>C	p.Lys432Thr	-	VUS	NA^a,b^	No
		*GGNBP2*	chr17:34913065	NM_024835.5:c.317C>G	p.Ser106Cys	-	LP	NA^a,b^	No
	NMTC_7	*NKD1*	chr16:50667583	NM_033119.5:c.1304T>C	p.Leu435Pro	-	VUS	NA^a,b^	No
		*ROBO1*	chr3:78763666	NM_002941.4:c.926T>C	p.Ile309Thr	-	VUS	NA^a,b^	No
		*MYH10*	chr17:8393804	NM_001256012.3:c.4738G>A	p.Glu1580Lys	-	VUS	NA^a,b^	No
		*TTC28*	chr22:28497190	NM_001145418.1:c.3386C>T	p.Ala1129Val	-	VUS	NA^a,b^	No
		*ZZEF1*	chr17:3937398	NM_015113.4:c.6495C>A	p.Asn2165Lys	-	VUS	NA^a,b^	No
		*CLIC6*	chr21:36043050	NM_053277.3:c.1363C>T	p.Leu455Phe	-	LP	NA^a,b^	No
		*CSMD2*	chr1:34068036	NM_052896.4:c.6649G>A	p.Gly2217Ser	-	LP	NA^a,b^	No
		*STK32A*	chr5:146741154	NM_001112724.2:c.637G>A	p.Ala213Thr	-	LP	NA^a,b^	No
		*TG*	chr8:134030213	NM_003235.5:c.6753G>T	p.Trp2251Cys	-	P	NA^a,b^	Yes
	NMTC_11	*NTRK1*	chr1:156843512	NM_002529.3:c.938T>A	p.Leu313Gln	-	VUS	NA^a,b^	Yes
		*TNKS*	chr8:9578001	NM_003747.3:c.1867C>A	p.Gln623Lys	-	VUS	NA^a,b^	No
		*ANKRD35*	chr1:145562755	NM_144698.5:c.2443T>C	p.Tyr815His	-	VUS	NA^a,b^	No
		*OR51M1*	chr11:5410964	NM_001004756.2:c.336G>C	p.Gln112His	-	VUS	NA^a,b^	No
		*PPP6R2*	chr22:50878140	NM_001242898.2:c.2139G>A	p.Trp713*	-	P	NA^a,b^	No
	NMTC_12	*FNTB*	chr14:65519983	NM_001202559.1:c.1166G>A	p.Trp389*	-	P	NA^a,b^	No
		*ITPR1*	chr3:4856888	NM_001168272.2:c.7808C>T	p.Thr2603Met	-	LP	NA^a,b^	No
		*PRKG1*	chr10:53921669	NM_006258.4:c.1022G>A	p.Gly341Glu	-	VUS	NA^a,b^	No
		*DENND2B*	chr11:8752138	NM_213618.2:c.697_699del	p.Ser233del	-	VUS	NA^a,b^	No
		*BMP1*	chr8:22064964	NM_006129.5:c.2510T>C	p.Met837Thr	-	VUS	NA^a,b^	Yes
		*THSD7A*	chr7:11521463	NM_015204.3:c.1969G>A	p.Gly657Arg	-	LP	NA^a,b^	No
		*INSC*	chr11:15243030	NM_001031853.4:c.968G>T	p.Gly323Val	-	LP	NA^a,b^	No
		*USH2A*	chr1:215848853	NM_206933.4:c.12400G>A	p.Ala4134Thr	-	LP	NA^a,b^	No
	NMTC_13	*MPPE1*	chr18:11886989	NM_023075.6:c.605del	p.Asp202Valfs*20	rs570653089	VUS	NA^a,b^	No
		*KTI12*	chr1:52498811	NM_138417.3:c.623T>A	p.Leu208His	-	VUS	NA^a,b^	No
		*BEAN1*	chr16:66511533	NM_001178020.3:c.360G>A	p.Trp120*	-	LP	NA^a,b^	No

**Table 3:** Possible interactions of thyroid cancer-associated genes harboring candidate variants. The interactions were predicted according to the ToppGene Suite tool.

Name	Genes	*p*-Value
MRE11 interactions	*NTRK1*; *HOOK3*; *BMP1*; *MSH6*	2.1 × 10^−7^
RAD50 interactions	*NTRK1*; *HOOK3*; *BMP1*; *MSH6*	2.9 × 10^−7^
NBN interactions	*HOOK3*; *BMP1*; *MSH6*	9.8 × 10^−6^
SMC2 interactions	*FOXM1*; *NTRK1*; *BMP1*	1.6 × 10^−5^

**Table 4:** Pathogenicity predictions for the identified segregating variants. SIFT scores range from 0.0 (deleterious (D)) to 1.0 (tolerated (T)), considering variants with a score from 0.0 to 0.05 as deleterious. PolyPhen-2 scores range from 0.0 (T) to 1.0 (D); variants with a score from 0.85 to 1 are more confidently predicted to be damaging. LoFtool provides a rank of genic intolerance and consequent susceptibility to disease based on the ratio of loss-of-function; the lower the LoFtool gene score percentile, the most intolerant the gene to functional variation (probably damaging (PD), damaging (D) and benign (B)). FATHMM score ≤ −1.5 was considered to be potentially pathogenic (P). Mutation Assessor scores below −1.5 were considered deleterious (M = medium, L = low and H = high). Mutation Taster predicts an alteration as one of four possible types: disease-causing (DC) (probably deleterious); DC automatic (deleterious), polymorphism (probably harmless); polymorphism automatic (harmless). The Combined Annotation Dependent Depletion score on the Phred scale (CADD phred) is shown for each variant, for which a score of 20 means that a variant is among the top 5% of deleterious variants in the human genome.

	Family ID	Gene	cDNA	SIFT	PolyPhen2	LoFtool	Mutation Assessor	Mutation Taster	FATHMM	CADD phred
Familial non-RET MTC	MTC_1	*PTPRS*	c.3548A>G	D (0.019)	P (1)	PD (0.317)	M (2.5)	DC (1)	T (0.52)	33
	MTC_3	*TBC1D4*	c.1862C>T	D (0.007)	PD (0.9)	B (0.771)	L (1.7)	DC (1)	T (0.95)	23.6
	MTC_4	*UBA7*	c.1232G>A	T (0.96)	B (0.002)	B (0.935)	L (1.2)	DC (1)	T(0.04)	20.7
		*NICN1*	c.56G>A	D (0)	PD (0.99)	PD (0.403)	M (2.11)	DC (1)	T (1.82)	29.6
		*MROH2A*	c.1736T>C	D (0.004)	---	---	---	DC (1)	T (0.56)	28.3
		*IL16*	c.1712_1718del	---	---	PD (0.492)	---	---	---	22.8
		*DDX51*	c.1262C>T	T (0.2)	B (0.44)	B (0.767)	L (1.83)	DC (1)	T (4.43)	22.3
		*CCDC134*	c.217del	---	---	PD (0.373)	---	---	---	33
		*ANKRD24*	c.2815G>C	D (0.047)	B (0.1)	B (0.852)	L (0.55)	DC (1)	T (1.32)	21.4
		*DNAH11*	c.1915C>T	---	---	---	---	DC (1)	---	34
		*MAPK12*	c.226C>T	D (0.01)	P (1)	B (0.778)	L (1.8)	DC (1)	T (−0.24)	32
	MTC_5	*ZNF19*	c.548A>G	D (0.001)	PD (0.66)	B (0.78)	M (3.15)	DC (1)	T (−0.27)	22.7
		*USP40*	c.3299C>A	T (0.063)	PD (0.718)	B (0.986)	M (2.71)	DC (1)	T (3.4)	23.3
		*MSH6*	c.3226C>T	D (0.023)	PD (0.99)	PD (0.112)	M (2.29)	DC (1)	D (−2.03)	29.3
		*DGKQ*	c.2296C>T	D (0.002)	P (1)	PD (0.367)	H (3.75)	DC (1)	T (1)	29.7
		*COL4A4*	c.286G>A	T (0.14)	PD (0.85)	D (−2.67)	L (1.105)	DC (0.62)	D (−3.38)	23.2
Familial NMTC	NMTC_1	*FOXM1*	c.176C>A	D (0.001)	PD (0.99)	PD (0.0154)	M (2.82)	DC (0.99)	D (−3.17)	26.3
		*EpCAM*	c.928A>T	D (0.001)	P (1)	---	M (2.95)	DC (0.95)	T (−0.9)	23.3
		*KRT39*	c.858del	---	---	---	---	---	---	33
		*BTBD16*	c.253G>T	---	---	---	---	DC (1)	---	36
	NMTC_2	*CACNA2D1*	c.3227A>C	D (0.004)	PD (0.99)	PD (0.258)	M (2.62)	DC (0.99)	T (3.14)	25.6
		*SHISA6*	c.1576C>T	D (0.017)	PD (0.99)	---	L (1.39)	DC (0.83)	---	25.1
		*AATK*	c.1342C>T	D (0)	P (1)	---	M (3.22)	DC (0.99)	D (−2.06)	25.4
	NMTC_4	*JMJD1C*	c.3743A>G	D (0)	PD (0.99)	PD (0.0285)	M (1.97)	DC (0.99)	T (0.03)	25.3
		*AGXT*	c.827T>G	D (0)	PD (0.99)	PD (0.0556)	H (4.23)	DC (1)	D (−2.56)	28
	NMTC_6	*HOOK3*	c.1448G>T	D (0.004)	PD (0.88)	PD (0.17)	M (2.1)	DC (1)	T (2.2)	24.6
		*RNF20*	c.1295A>C	D (0.002)	PD (0.9)	B (0.764)	M (2.01)	DC (0.99)	T (1.19)	23.1
		*GGNBP2*	c.317C>G	--	PD (0.98)	PD (0.157)	M (2.25)	DC (0.99)	---	26.3
	NMTC_7	*NKD1*	c.1304T>C	D (0)	PD (0.99)	PD (0.0705)	L (1.38)	DC (1)	T(−1.09)	24.3
		*ROBO1*	c.926T>C	D (0.001)	PD (0.83)	B (0.687)	M (2.155)	DC (0.99)	T (−0.4)	26.6
		*MYH10*	c.4738G>A	D (0.013)	PD (0.86)	---	H (3.815)	DC (1)	D (−2.04)	26.6
		*TTC28*	c.3386C>T	D (0.013)	PD (0.83)	---	L (1.61)	DC (1)	T (−1.01)	25.4
		*ZZEF1*	c.6495C>A	T (0.1)	B (0.001)	PD (0.55)	L (0.345)	DC (0.83)	T (2.17)	20.2
		*CLIC6*	c.1363C>T	D (0)	PD (0.99)	---	L (1.9)	DC (1)	T (0.9)	29.9
		*CSMD2*	c.6649G>A	D (0)	PD (0.99)	PD (0.316)	M (2.745)	DC (1)	T 1.22)	31
		*STK32A*	c.637G>A	D (0)	PD (0.99)	B (0.87)	L (0.99)	DC (0.99)	T (1.83)	25.8
		*TG*	c.6753G>T	D (0)	PD (0.99)	B (0.858)	H (4.57)	DC (1)	T (−0.97)	32
	NMTC_11	*NTRK1*	c.938T>A	D (0.001)	PD (0.98)	PD (0.0395)	M (2.285)	DC (1)	T (1.44)	27.6
		*TNKS*	c.1867C>A	D (0.024)	B (0.43)	PD (0.41)	M (2.545)	DC (1)	T (0.67)	23.8
		*ANKRD35*	c.2443T>C	---	---	B (0.99)	---	---	---	24.2
		*OR51M1*	c.336G>C	D (0)	---	PD (0.377)	H (3.73)	DC (1)	T (7.19)	24.6
		*PPP6R2*	c.2139G>A	---	---	---	---	DC (1)	---	47
	NMTC_12	*FNTB*	c.1166G>A	---	---	---	---	DC (1)	---	45
		*ITPR1*	c.7808C>T	D (0)	PD (0.98)	PD (0.0141)	M (3.013)	DC (1)	T (0.81)	30
		*PRKG1*	c.1022G>A	D (0.001)	PD (0.98)	PD (0.0367)	L (1.53)	DC (1)	T (1.5)	28
		*DENND2B*	c.697_699del	---	---	PD (0.39)	---	---	---	21.6
		*BMP1*	c.2510T>C	D (0.001)	PD (0.98)	B (0.687)	M (2.99)	DC (1)	T (2.05)	26.8
		*THSD7A*	c.1969G>A	D (0)	P (1)	---	H (3.865)	DC (1)	D (−1.75)	31
		*INSC*	c.968G>T	D (0)	PD (0.99)	B (0.687)	L (1.04)	DC (1)	T (0.93)	26.9
		*USH2A*	c.12400G>A	D (0)	PD (0.99)	B (0.92)	M (3.425)	DC (1)	T (−1.02)	28.5
	NMTC_13	*MPPE1*	c.605del	---	---	B (0.93)	---	---	---	31
		*KTI12*	c.623T>A	D (0.004)	P (1)	---	M (3.15)	DC (0.99)	T (1.23)	25
		*BEAN1*	c.360G>A	---	---	---	---	DC (1)	---	37

**Table 5:** Prediction of the structural and functional consequences of variants affecting residues buried within the core of a domain of the protein through Project HOPE. (+ve): Positive charge; (−ve): negative charge; (0): neutral charge.

	ID	SNVs	Gene	Wild-Type Size and (Charge)	Mutant Size and (Charge)	Characteristics & Features
Familial non-RET MTC	MTC_4	R411K	*UBA7*	Large & (0)	Small & (0)	The mutation is located within a stretch of residues annotated as a special region: 2 approximate repeats
						Loss of interaction: High
						Protean folding: Affected
		L579P	*MROH2A*	Large & (0)	Small & (0)	The mutation is located within a stretch of residues that is repeated in the protein, HEAT 7
						Loss of interaction: High
						Protean folding: Affected
		P421L	*DDX51*	Small & (0)	Large & (0)	The mutation is located within a domain, Helicase ATP-binding
						Loss of interaction: High
						Protean folding: Affected
		R76C	*MAPK12*	Large & (+ve)	Small & (0)	The mutation is located within a domain, Protein kinase
						Hydrophobicity: High
						Loss of interaction: High
						Protean folding: Affected
	MTC_5	H183R	*ZNF19*	Small & (0)	Large & (+ve)	Mutant residue disturb the interaction with the metal-ion: “Zinc”
						Loss of interaction: High
						Protean folding: Affected
		D96N	*COL4A4*	Small & (−ve)	Small & (0)	The mutation is located within a special motif: Cell attachment site and Triple-helical region
						Loss of interaction: High
						Protean folding: Affected
Familial NMTC	NMTC_2	Q1076P	*CACNA2D1*	Small & (0)	Large & (0)	The residue is located in a region annotated as a transmembrane domain
						Loss of interaction: High
						Protean folding: Affected
						Hydrophobicity: High
	NMTC_7	I309T	*ROBO1*	Large	Small	The mutation is located within a domain, Ig-like C2-type 3
						Loss of interaction: High
						Protean folding: Affected
						Hydrophobicity: Lost
		G2217S	*CSMD2*	Small	Large	The mutation is located within a domain, CUB 13
						Loss of interaction: High
						Protean folding: Affected
		A213T	*STK32A*	Small	Large	The mutation is located within a domain, Protein kinase
						Loss of interaction: High
						Protean folding: Affected
						Hydrophobicity: Lost
		W2251C	*TG*	Large	Small	The mutation is located within a special region: Cholinesterase-like (ChEL)
						Loss of interaction: High
						Protean folding: Affected
	NMTC_11	L313Q	*NTRK1*	Small	Large	The mutation is located within a domain, Ig-like C2-type 2
						Loss of interaction: High
						Protean folding: Affected
						Hydrophobicity: Lost
		Q623K	*TNKS*	Small & (0)	Large & (+ve)	The mutation is located within a stretch of residues that is repeated in the protein, ANK 12
						Loss of interaction: High
						Protean folding: Affected
		Q112H	*OR51M1*	Small	Large	The residue is located in a region annotated as a transmembrane domain
						Loss of interaction: High
						Protean folding: Affected
	NMTC_12	G341E	*PRKG1*	Small & (0)	Large & (−ve)	The mutation is located within a domain, GMP-binding, low affinity
						Loss of interaction: High
						Protean folding: Affected
		M837T	*BMP1*	Large	Small	The mutation is located within a domain, CUB 4
						Loss of interaction: High
						Protean folding: Affected
						Hydrophobicity: Lost
		G657R	*THSD7A*	Small & (0)	Large & (+ve)	The mutation is located within a domain, TSP type-1 7
						Loss of interaction: High
						Protean folding: Affected
		A4134T	*USH2A*	Large & (+ve)	Small & (0)	The mutation is located within a domain, Laminin EGF-like 3
						Loss of interaction: High
						Protean folding: Affected
						Hydrophobicity: High

## Data Availability

The data that support the findings of this study are available from the corresponding authors upon reasonable request.

## Supplementary Materials

The supporting information can be downloaded at: https://www.mdpi.com/article/10.3390/ijms24097843/s1.
