# Supplementary material for: Identification of Novel Candidate Genes for Familial Thyroid Cancer by Whole Exome Sequencing
Source: Int J Mol Sci. 2023 Apr 25;24(9):7843. doi: 10.3390/ijms24097843 (PMC10178269; doi:10.3390/ijms24097843)
Supplement: Supplementary file 1 [file ijms-24-07843-s001.zip › supplementary figure and table caption.pdf]

Table S1. Prediction of structural and functional consequences of variants through Project HOPE.,

Table S2. Sequences of the forward and reverse primers,

Figure S1 and S2. Pedigree charts of the Spanish families included in this study.
