# Supplementary material for: Identification of Novel Candidate Genes for Familial Thyroid Cancer by Whole Exome Sequencing
Source: Int J Mol Sci. 2023 Apr 25;24(9):7843. doi: 10.3390/ijms24097843 (PMC10178269; doi:10.3390/ijms24097843)
Supplement: Supplementary file 1 [file ijms-24-07843-s001.zip › Table_S1.pdf]

	ID	SNVs	Gene	Wild-Type Size and (Charge)	Mutant Size and (Charge)	Characteristics & Features
Familial non-*RET* MTC	**MTC_1**	E1183G	*PTPRS*	Large & (+ve)	Small & (0)	Hydrophobicity: High<br>Loss of interaction: High<br>Protean folding: Affected
	**MTC_3**	S621L	*TBC1D4*	Small & (0)	Large & (0)	Hydrophobicity: High<br>Loss of interaction: High<br>Protean folding: Affected
	**MTC_4**	R411K	*UBA7*	Large & (0)	Small & (0)	The mutation is located within a stretch of residues annotated as a special region: 2 approximate repeats<br>Loss of interaction: High<br>Protean folding: Affected
		G19D	*NICN1*	Small & (0)	Large & (0)	Hydrophobicity: Lost<br>Loss of interaction: High<br>Protean folding: Affected
		L579P	*MROH2A*	Large & (0)	Small & (0)	The mutation is located within a stretch of residues that is repeated in the protein, HEAT 7<br>Loss of interaction: High<br>Protean folding: Affected
		E571GfsTer7	*IL16*			
		P421L	*DDX51*	Small & (0)	Large & (0)	The mutation is located within a domain, Helicase ATP-binding<br>Loss of interaction: High<br>Protean folding: Affected
		L73SfsTer29	*CCDC134*			
		E939Q	*ANKRD24*	Large & (-ve)	Large & (0)	Loss of interaction: High<br>Protean folding: Affected
		Q639Ter	*DNAH11*			
		R76C	*MAPK12*	Large & (+ve)	Small & (0)	The mutation is located within a domain, Protein kinase;<br>Hydrophobicity: High<br>Loss of interaction: High<br>Protean folding: Affected
	**MTC_5**	H183R	*ZNF19*	Small & (0)	Large & (+ve)	Mutant residue disturb the interaction with the metal-ion: "Zinc"<br>Loss of interaction: High<br>Protean folding: Affected
		A1111D	*USP40*	Small	Large	Hydrophobicity: Lost<br>Protean folding: Affected
		R1076C	*MSH6*	Large & (+ve)	Small & (0)	Loss of interaction: High<br>Protean folding: Affected<br>Hydrophobicity: High
		P766S	*DGKQ*	Large	Small	Loss of interaction: High<br>Protean folding: Affected<br>Hydrophobicity: Lost
		D96N	*COL4A4*	Small & (-ve)	Small & (0)	The mutation is located within a special motif: Cell attachment site and Triple-helical region<br>Loss of interaction: High<br>Protean folding: Affected
Familial NMTC	**NMTC _1**	P59Q	*FOXM1*	Small & (0)	Large & (-ve)	Loss of interaction: High<br>Protean folding: Affected<br>Hydrophobicity: Lost
		R310W	*EpCAM*	Small & (+ve)	Large & (0)	Loss of interaction: High<br>Protean folding: Affected<br>Hydrophobicity: High
		W286SfsTer6	*KRT39*			
		E85Ter	*BTBD16*			
	**NMTC _2**	Q1076P	*CACNA2D1*	Small & (0)	Large & (0)	The residue is located in a region annotated as a transmembrane domain<br>Loss of interaction: High<br>Protean folding: Affected<br>Hydrophobicity: High
		H526Y	*SHISA6*	Small	Large	Loss of interaction: High<br>Protean folding: Affected<br>Hydrophobicity: High
		P448S	*AATK*	Large & (0)	Small & (0)	Loss of interaction: High<br>Protean folding: Affected
	**NMTC _4**	Q1248R	*JMJD1C*	Small & (0)	Large & (+ve)	Loss of interaction: High<br>Protean folding: Affected
		L276R	*AGXT*	Small & (0)	Large & (+ve)	Loss of interaction: High<br>Protean folding: Affected<br>Hydrophobicity: Lost
	**NMTC_6**	G483V	*HOOK3*	Small & (0)	Large & (0)	Loss of interaction: High<br>Protean folding: Affected<br>Hydrophobicity: High
		K432T	*RNF20*	Large & (+ve)	Small & (0)	Loss of interaction: High<br>Protean folding: Affected<br>Hydrophobicity: High
		S106C	*GGNBP2*	Small	Small	Loss of interaction: High<br>Protean folding: Affected<br>Hydrophobicity: High
	**NMTC _7**	L435P	*NKD1*	Large	Small	Loss of interaction: High
		I309T	*ROBO1*	Large	Small	The mutation is located within a domain, Ig-like C2-type 3<br>Loss of interaction: High<br>Protean folding: Affected<br>Hydrophobicity: Lost
		E1580K	*MYH10*	Small & (0)	Large & (+ve)	Loss of interaction: High<br>Protean folding: Affected
		A1129V	*TTC28*	Small	Large	Loss of interaction: High<br>Protean folding: Affected
		N2165K	*ZZEF1*	Small & (0)	Large & (+ve)	Loss of interaction: High<br>Protean folding: Affected
		L455F	*CLIC6*	Small	Large	Loss of interaction: High<br>Protean folding: Affected
		G2217S	*CSMD2*	Small	Large	The mutation is located within a domain, CUB 13<br>Loss of interaction: High<br>Protean folding: Affected
		A213T	*STK32A*	Small	Large	The mutation is located within a domain, Protein kinase<br>Loss of interaction: High<br>Protean folding: Affected<br>Hydrophobicity: Lost
		W2251C	*TG*	Large	Small	The mutation is located within a special region: Cholinesterase-like (ChEL)<br>Loss of interaction: High<br>Protean folding: Affected
	**NMTC_11**	L313Q	*NTRK1*	Small	Large	The mutation is located within a domain, Ig-like C2-type 2<br>Loss of interaction: High<br>Protean folding: Affected<br>Hydrophobicity: Lost
		Q623K	*TNKS*	Small & (0)	Large & (+ve)	The mutation is located within a stretch of residues that is repeated in the protein, ANK 12<br>Loss of interaction: High<br>Protean folding: Affected
		Y815H	*ANKRD35*	Small	Large	Loss of interaction: High<br>Protean folding: Affected<br>Hydrophobicity: Lost
		Q112H	*OR51M1*	Small	Large	The residue is located in a region annotated as a transmembrane domain<br>Loss of interaction: High<br>Protean folding: Affected
		W713Ter	*PPP6R2*			
	**NMTC_12**	W389Ter	*FNTB*			
		T2603M	*ITPR1*	Small	Large	Loss of interaction: High<br>Protean folding: Affected<br>Hydrophobicity: High
		G341E	*PRKG1*	Small & (0)	Large & (-ve)	The mutation is located within a domain, GMP-binding, low affinity<br>Loss of interaction: High<br>Protean folding: Affected
		S233del	*DENND2B*			
		M837T	*BMP1*	Large	Small	The mutation is located within a domain, CUB 4<br>Loss of interaction: High<br>Protean folding: Affected<br>Hydrophobicity: Lost
		G657R	*THSD7A*	Small & (0)	Large & (+ve)	The mutation is located within a domain, TSP type-1 7<br>Loss of interaction: High<br>Protean folding: Affected
		G323V	*INSC*	Small	Large	Loss of interaction: High<br>Protean folding: Affected<br>Hydrophobicity: High
		A4134T	*USH2A*	Large & (+ve)	Small & (0)	The mutation is located within a domain, Laminin EGF-like 3<br>Loss of interaction: High<br>Protean folding: Affected<br>Hydrophobicity: High
	**NMTC_13**	D202VfsTer20	*MPPE1*			
		L208H	*KTI12*	Small	Large	Loss of interaction: High<br>Protean folding: Affected<br>Hydrophobicity: Lost
		W120Ter	*BEAN1*			
