# Supplementary material for: Identification of Novel Candidate Genes for Familial Thyroid Cancer by Whole Exome Sequencing
Source: Int J Mol Sci. 2023 Apr 25;24(9):7843. doi: 10.3390/ijms24097843 (PMC10178269; doi:10.3390/ijms24097843)
Supplement: Supplementary file 1 [file ijms-24-07843-s001.zip › Table_S2.pdf]

	Family ID	Chr	Gene	Forward Primer	Reverse Primer	Optimal Annealing Temperature
**Familial non-*RET* MTC**	**MTC_1**	chr19	*PTPRS*	5′AGGCCTGAGTCAGCAACTGT	5′ TGTAGCCGTGAGATGTCCTG	57 °C
	**MTC_3**	chr13	*TBC1D4*	5′TGACTCAGGAGGAAGATGAG	5′TCATTCTGGAGAGGGTCAG	58 °C
	**MTC_4**	chr3	*UBA7*	5 TGATGCCCTCGATTGTCTTC	5′TTGGCAGGAACAGCCTTAG	52 °C
		chr3	*NICN1*	5′CCCGGGATTTGTAGTTCTTG	5′GCAGAGAACAAGACAGGAAG	53 °C
		chr2	*MROH2A*	5′GCCAACCCATTCTCCATTCT	5′GCACCTCAGCTTCCTCATAC	60 °C
		chr15	*IL16*	5′TGGACATCAGCACACACAG	5′CCAGAAGAGCATGAGGAGTTC	55 °C
		chr12	*DDX51*	5′CCTTCCTTAATGCCCAGAGATAA	5′CCCAACAGGAAAGGCATACT	60 °C
		chr22	*CCDC134*	5′TTCTGCCCAGACAAGAAGATG	5′CCGTTAGGGAACTCCTGTTTAC	55 °C
		chr19	*ANKRD24*	5′GAGCTGGAGGTTCTGCGG	5′TCTGCGCTGGTCTTCTCATG	63 °C
		chr7	*DNAH11*	5′GTTCCCTTTCTTCCTCTTCAC	5′ATGTCACCCGTACCATTAGA	56 °C
		chr22	*MAPK12*	5′GCTAAGGTGGCCATCAAGAA	5′TCTCCCAAGGAGAGACAGG	58 °C
	**MTC_5**	chr16	*ZNF19*	5′GTCCCTCAGAGAACTGACTT	5′CCACACTCCTCACACTGATA	60 °C
		chr2	*USP40*	5′AAGCTGGAGGAGAAGCTTTG	5′CCTCATTGCTATGCCGGTAA	60 °C
		chr2	*MSH6*	5′GCACTTCTATGGTCCAGATG	5′TCTTCGTAATGCAAGGATGG	56 °C
		chr4	*DGKQ*	5′GTCCTTGCTCTTGTCTCTG	5′GGTACCTGCTTGTGAACTT	53 °C
		chr2	COL4A4	5′GTCTTCTGTAGGCCTGAATG	5′CTAGCCTGAGTGACAGAGTA	55 °C
**Familial NMTC**	**NMTC _1**	chr12	*FOXM1*	5′CCCAACAGGAGTCTAATCAAG	5′CACAGCTGATGAGGATGAAT	53 °C
		chr2	EpCAM	5′CTAGCTTACCTCCAACTTGC	5′GAAAGGCAGCTTTCAATCAC	55 °C
		chr17	KRT39	5′CCCTTCTGCTGACCTAAAC	5′ACAGCCAGAGACTGAGAATA	55 °C
		chr10	BTBD16	5′CACTTCACTGCTTCCTTTCT	5′GATAACACTGTGGAGCCTTAC	55 °C
	**NMTC _2**	chr7	*CACNA2D1*	5′GGATTACTCTGAAAGATGTGTTACT	5′ATAGCTGACCCTACGTTACTG	58 °C
		chr17	SHISA6	5′ATCCCAGTGCCTCCAATAA	5′GAGAGTGGCAAGGCTAGAA	55 °C
		chr17	AATK	5′CGAAGCAGAGGAGGAGTTT	5′GCCTCCCACTTGTACTCAA	60 °C
	**NMTC _4**	chr10	*JMJD1C*	5′TTTCACCCACCAATCCATC	5′CCACATCTCCGTCAAACTC	55 °C
		chr2	AGXT	5′TCCCGGTCCAAAGTTCT	5′ACCAGGGATGGTTCCTT	55 °C
	**NMTC_6**	chr8	*HOOK3*	5′ TGTAAGAGTGCTTAGCTGGATTT	5′TCCCAAAGTGCTGGGATTAC	56 °C
		chr9	*RNF20*	5′GGTTTCATTACGTATAGATTGAACTGG	5′CTACAGCATGGAGTGAGAATCAT	60 °C
		chr17	GGNBP2	5′GAAGTCCTGAGTGCACTTTC	5′CACTGTCACCAAGCTGTTT	55 °C
	**NMTC _7**	chr16	*NKD1*	5′GCACAAGAAGCACAAGCAC	5′GGTGGTAATGGTGGTGATGT	55 °C
		chr3	*ROBO1*	5′CCAGCTGACTTAGAGGAATAC	5′CTTTCCCAATGCCTACCTT	52 °C
		chr17	*MYH10*	5′CGGTCAACTCTTTCCTGATG	5′TTTGCAGGTCTCTCTCGAA	53 °C
		chr22	*TTC28*	5′CCTTCAGGACTCCTTAGGATA	5′GTAGAAGTCAGTGCCTCTTG	53 °C
		chr17	*ZZEF1*	5′CCACCTGAATGAAACCTACCA	5′ATAAACACCAAGGTGGGATAGAG	54 °C
		chr21	CLIC6	5′CAGCTCAGCAACCACCT	5′AGATGACCAAGACCTCGAATG	56 °C
		chr1	CSMD2	5′GAACATCACTTCTTCCAACGG	5′CCCAGTAATGCTCCTTTCTAATTG	56 °C
		chr5	STK32A	5′CCTTTGGAGGAACATGTTGAGA	5′CTGCTTGGATCTTAGCAAACAATAC	56 °C
		chr8	TG	5′TGTACCTTCTGTGCCCATTTC	5′CTCTGCTACTGAGTCCCATTTG	56 °C
	**NMTC_11**	chr1	*NTRK1*	5′CTCCTGCTGTTGCTCTTTC	5′TGTAGTTGCCGTTGTTGAC	58 °C
		chr8	*TNKS*	5′ ATGTGCCCACGCAATGA	5′ATTTCAGCAGGGATGGAAGAG	58 °C
		chr1	ANKRD35	5′GAAGCTGGAGGAAGAGCTG	5′TTTCCAACAGAGCCTTTAGC	56 °C
		chr11	OR51M1	5′ATTAAGACCAACCCTCGTCTG	5′ACAATTAGGCCTGCTCTGAC	56 °C
		chr22	PPP6R2	5′GACGGCAGTGTTTGATGAG	5′ATACAAGGACGAGGACAGG	55 °C
	**NMTC_12**	chr14	*FNTB*	5′CCCAGGTGAGAAAGTGGAATTA	5′ACACTCACTTGCCAGGTTTAT	54 °C
		chr3	*ITPR1*	5′CCCTGTTTGCTGCTAGAGTTA	5′CATCACACCCTCGCAGTATC	55 °C
		chr10	*PRKG1*	5′CTAGGTTGCCATTGCTACTATTT	5′GTTGTTTCCTGTCAGAGTGTTG	54 °C
		chr11	*DENND2B*	5′GAGCATGTGTGGAGAGAAG	5′TAGAAAGAGTCCCGGACAG	60 °C
		chr8	*BMP1*	5′TGTGCCTACGACCACCTA	5′TCAGTGAACACTCCCACAAC	55 °C
		chr7	THSD7A	5′CCCAGGAGAAGAAGTTGACAG	5′GAACCTAGATGGCGTCTGAAG	56 °C
		chr11	INSC	5′CAGAGCTGGCTTCCATGTAG	5′TACTGAGGTGCTGGGTGA	56 °C
		chr1	USH2A	5′CAAAGAAATTGTATGCTGCAAG	5′GGGCTTCATCTGTCCAC	53 °C
	**NMTC_13**	chr18	MPPE1	5′TTTCATGGGAACAGTAGGGAAA	5′AGCAACACGGGACAGAAG	55 °C
		chr1	KTI12	5′GAACTGGAGCGAGAAGAATC	5′GTTCTCAAACAGGGCAGAG	56 °C
		chr16	BEAN1	5′GGTAGACCTACAGACCCATAA	5′TACCCTGGTGGAGAATCTG	56 °C
