# Supplementary figures and images for: Identification of Novel Candidate Genes for Familial Thyroid Cancer by Whole Exome Sequencing
Source: Int J Mol Sci. 2023 Apr 25;24(9):7843. doi: 10.3390/ijms24097843 (PMC10178269; doi:10.3390/ijms24097843)

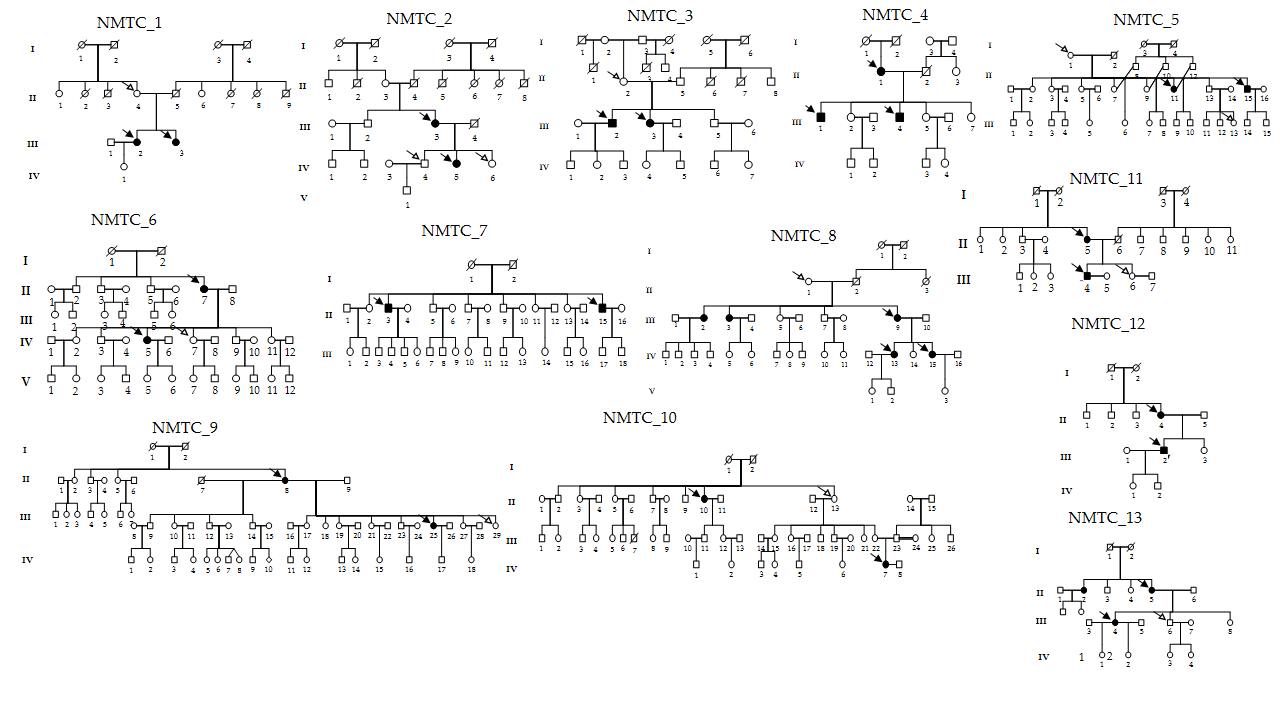

Supplement: Supplementary file 1 [file ijms-24-07843-s001.zip › Figure_S1.tif]

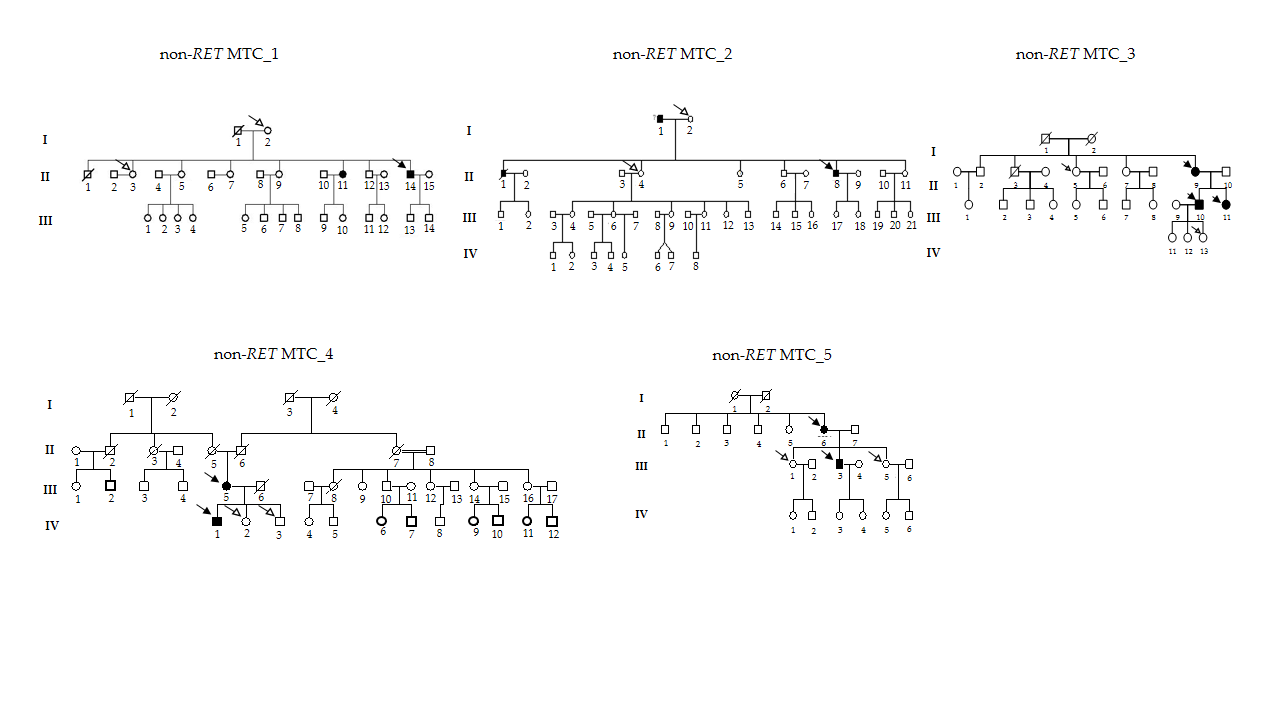

Supplement: Supplementary file 1 [file ijms-24-07843-s001.zip › Figure_S2.tif]
